# CovidLife: a resource to understand mental health, well-being and behaviour during the COVID-19 pandemic in the UK

**DOI:** 10.12688/wellcomeopenres.16987.1

**Published:** 2021-07-07

**Authors:** Chloe Fawns-Ritchie, Drew M. Altschul, Archie Campbell, Charlotte Huggins, Clifford Nangle, Rebecca Dawson, Rachel Edwards, Robin Flaig, Louise Hartley, Christie Levein, Daniel L. McCartney, David Bell, Elaine Douglas, Ian J. Deary, Caroline Hayward, Riccardo E. Marioni, Andrew M. McIntosh, Cathie Sudlow, David J. Porteous

**Affiliations:** 1 Centre for Genomic and Experimental Medicine, Institute of Genetics and Cancer, University of Edinburgh, Edinburgh, EH4 2XU, UK; 2 Department of Psychology, University of Edinburgh, Edinburgh, EH8 9JZ, UK; 3 Centre for Medical Informatics, Usher Institute, University of Edinburgh, Edinburgh, EH16 4UX, UK; 4 MRC Human Genetics Unit, Institute of Genetics and Cancer, University of Edinburgh, Edinburgh, EH4 2XU, UK; 5 Division of Economics, Stirling Management School, University of Stirling, Stirling, FK9 4LA, UK; 6 Faculty of Social Sciences, University of Stirling, Stirling, FK9 4LA, UK; 7 Division of Psychiatry, University of Edinburgh, Edinburgh, EH10 5HF, UK

**Keywords:** COVID-19, psychological, mental health, well-being, longitudinal study, observational study

## Abstract

CovidLife is a longitudinal observational study designed to investigate the impact of the COVID-19 pandemic on mental health, well-being and behaviour in adults living in the UK. In total, 18,518 participants (mean age = 56.43, SD = 14.35) completed the first CovidLife questionnaire (CovidLife1) between April and June 2020. To date, participants have completed two follow-up assessments. CovidLife2 took place between July and August 2020 (n = 11,319), and CovidLife3 took place in February 2021 (n = 10,386). A range of social and psychological measures were administered at each wave including assessments of anxiety, depression, well-being, loneliness and isolation. Information on sociodemographic, health, and economic circumstances was also collected. Questions also assessed information on COVID-19 infections and symptoms, compliance to COVID-19 restrictions, and opinions on the UK and Scottish Governments’ handling of the pandemic.

CovidLife includes a subsample of 4,847 participants from the Generation Scotland cohort (N~24,000, collected 2006–2011); a well-characterised cohort of families in Scotland with pre-pandemic data on mental health, physical health, lifestyle, and socioeconomic factors, along with biochemical and genomic data derived from biological samples. These participants also consented to their study data being linked to Scottish health records.

CovidLife and Generation Scotland data can be accessed and used by external researchers following approval from the Generation Scotland Access Committee. CovidLife can be used to investigate mental health, well-being and behaviour during COVID-19; how these vary according to sociodemographic, health and economic circumstances; and how these change over time. The Generation Scotland subsample with pre-pandemic data and linkage to health records can be used to investigate the predictors of health and well-being during COVID-19 and the future health consequences of the COVID-19 pandemic.

## Introduction

The COVID-19 pandemic and the mitigation measures to reduce its spread resulted in substantial changes to everyday life in the UK and globally. Early 2020 saw rising COVID-19 infection rates and deaths in the UK and a national lockdown was announced on 23 ^rd^ March 2020, requiring everyone to stay at home at all times, with very limited exceptions.

Throughout 2020 and 2021, COVID-19 mitigation measures have eased and tightened as the number of cases, hospitalisations and deaths have reduced and increased. However, some form of restrictions have remained in place since the first stay at home order was announced on 23 ^rd^ March 2020. While mitigation measures successfully reduce the number of COVID-19 infections, hospitalisations, and deaths ^1, 2^, they may negatively impact mental health and well-being. Concerns about the impact of COVID-19 on mental health led leading health journals to call for research into the effect of COVID-19 on mental health ^3, 4^.

Longitudinal studies have reported that rates of psychological distress increased during the early stages of the COVID-19 pandemic ^5– 8^. A rapid review and meta-analysis of 12 longitudinal studies with pre-pandemic data found small but significant increases in the rates of depressive symptoms (Hedge’s *g* = 0.15, SE = 0.07, 95% CI 0.01 to 0.30, p = .037) and anxiety ( *g* = 0.17, SE = 0.05, 95% CI 0.07 to 0.27, p < .001) during lockdown (January to June 2020), when compared to before the pandemic ^9^.

The psychological impact of the COVID-19 mitigation measures may not affect everyone equally. Female participants and younger adults have consistently shown higher rates of psychological distress and loneliness during the COVID-19 pandemic, and show larger increases in psychological distress compared to pre-pandemic levels ^5, 6, 8, 10^. Different sociodemographic characteristics have been associated with higher rates of psychological distress during COVID-19, including people from Asian minority ethnic groups ^6, 8^, people with pre-existing health conditions ^6, 11^, caregivers ^12^, people who are unemployed or economically inactive ^8^, those who report experiencing abuse or low social support ^11^, those with children under five years ^8^, and parents doing 20+ hours of childcare or home schooling a week ^13^.

The degree to which COVID-19 and its mitigation measures affect the daily lives of people in the UK may change over time. CovidLife is a longitudinal study that uses online questionnaires to assess the psychological impact of COVID-19 during the first lockdown and how this changes over time as the pandemic progresses. Sociodemographic, economic and health indicators were measured to determine the cross-sectional and longitudinal correlates of mental health, well-being and behaviour during COVID-19. We also measured the degree of adherence and attitudes towards the mitigation measures to understand their associations with mental health and behaviour.

CovidLife consists of 18,518 adults aged 18 years and older resident in the UK who completed the first (baseline) online questionnaire between April and June 2020. In addition to recruiting anyone resident in the UK, invitations were sent to members of the Generation Scotland cohort ^14^, a well characterised cohort of Scottish families with data on health and lifestyle collected between 9 and 14 years before the onset of the COVID-19 pandemic. Biological samples were also collected from Generation Scotland participants and consent for linkage to Scottish health records was obtained ^14, 15^. This subsample can therefore be used to investigate the health, lifestyle and biological predictors of mental health, well-being and behaviour during the COVID-19 pandemic.

CovidLife was designed to be a resource for researchers investigating the impact of the COVID-19 pandemic on health and well-being. Researchers can apply to access the CovidLife data. This paper describes the development of the CovidLife questionnaires, characterises the cohort, and describes the data collected in the first three waves.

## Methods

### Development of the CovidLife questionnaire

The CovidLife questionnaires were developed by the Generation Scotland Team using Qualtrics survey software, a survey development tool ^16^. Data collection was limited to remote online assessment due to the COVID-19 restrictions. Online data collection also enabled quick data capture during the COVID-19 pandemic. The questionnaires were designed to be suitable for completion on various devices, including desktop computers, tablets and smartphones.

### Questionnaire content

The questionnaire was developed primarily to understand how participants were feeling and behaving during the COVID-19 pandemic. The topics assessed in the first three CovidLife questionnaires are shown in Table 1. All three CovidLife questionnaires administered to date are available in the *Extended data* ^17^ (also available at www.ed.ac.uk/generation-scotland/for-researchers/covidlife).

**Table 1.** Topics assessed in CovidLife1, CovidLife2, and CovidLife3.

	CovidLife1	CovidLife2	CovidLife3
***Sociodemographic***			
Date of birth	X		
Sex and gender identity	X		
Country of residence	X		
Ethnicity	X		
Household make-up	X	X	X
Relationship status	X		X
Educational qualifications	X		
Accommodation type and tenure	X		
Caring responsibilities	X		
Disabilities	X		
***Health***			
Self-reported health	X		X
Self-reported height and weight	X		X
Self-reported health conditions	X		
Self-reported mental health conditions	X		
Currently pregnant	X		X
Shielding status	X		
COVID-19 status (suspected, tested positive)	X	X	X
COVID-19 symptoms	X	X	X
COVID-19 testing			X
Long-COVID			X
COVID-19 status of others in household	X	X	
Had COVID-19 vaccine			X
Smoking	X		
Alcohol consumption	X		
Physical activity	X		X
Diet	X		X
Sleep	X		X
Postponed healthcare		X	
COVID-19 vaccine hesitancy			X
Vaccine hesitancy			X
***Psychological measures***			
Depressive symptoms	X	X	X
Anxiety	X	X	X
Well-being	X	X	X
Life satisfaction	X	X	X
Perceived Stress		X	X
Resilience		X	
Personality	X		
Health literacy	X		
Worries during COVID-19	X	X	X
***Social support and relationships***			
Loneliness	X	X	X
Isolation	X	X	X
Whether someone could provide support if had COVID-19	X		
Quality of relationships with family and friends	X	X	X
Keeping in touch with family and friends	X	X	X
Childcare during COVID-19		X	
Home schooling during COVID-19		X	
Pets	X		
Social media use	X	X	X
Know anyone who has died from COVID-19		X	X
***Employment and financial circumstances***			
Current employment status	X	X	X
Employment status before COVID-19	X	X	
Type of work	X		
Whether furloughed	X	X	X
Working from home	X	X	X
Key worker status	X	X	
Confidence in employment post-COVID-19	X		
PPE available at work		X	X
Applied for/received support for self- employed	X	X	X
Spouse/partner employment		X	
Household income	X		
Receiving benefits	X	X	X
Change to financial situation	X	X	X
***COVID-19 knowledge, attitudes and behaviour***			
Perceived public health threat of COVID-19	X		X
Attitudes towards following Government COVID-19 restrictions	X	X	X
Adherence to COVID-19 restrictions	X	X	X
Knowledge of COVID-19 restrictions	X	X	X
Sources of COVID-19 information	X		X
Confidence in Government to prevent further spread of COVID-19	X	X	X
Confidence in NHS to cope with COVID-19	X		X
Impact of COVID-19/COVID-19 restrictions	X	X	X
New pastimes during COVID-19	X		
Confidence in test, track and trace systems		X	
Using test, track and trace systems			X
Instructed to self-isolate by contact tracers			X
Changes in public transport use		X	
Trust in medicine and science			X

Validated scales were used to assess a range of psychological variables including anxiety and depressive symptoms, well-being, personality, stress and resilience ( Table 2). Selected items from other COVID-19 questionnaires were also used ( Table 2). To assess COVID-19 knowledge and attitudes, questions from the Chicago COVID-19 Comorbidities survey ^18^ were adapted for use with UK participants. The second CovidLife questionnaire included a section on childcare during COVID-19, using questions adapted from the Gender Division of Childcare during the COVID-19 Pandemic survey ^19^. Where possible, questions aligned with those administered in other longitudinal population studies. Many of the questions used in CovidLife also form part of the Wellcome Trust’s COVID-19 questionnaire. The Wellcome Trust’s COVID-19 questionnaire was co-developed by Generation Scotland as part of the Wellcome Trust Longitudinal Population Study COVID-19 Steering Group and Secretariat and is freely available to population health researchers to use to investigate the impact of the COVID-19 pandemic ( www.bristol.ac.uk/alspac/researchers/wellcome-covid-19/). Using the same or similar items as other studies enables harmonisation, replication and collaboration with other population health studies. Where necessary, new questions were developed to understand participants’ circumstances during the COVID-19 pandemic.

**Table 2.** Validated scales and questionnaires used in CovidLife.

Topic	Measure
***Validated scales***	
Anxiety symptoms	Generalised Anxiety Disorder 7-item questionnaire (GAD-7) ^20^
Depressive symptoms	Patient Health Questionnaire 9-item version (PHQ-9) ^21^
Well-being	Short Warwick-Edinburgh Mental Well-being Scale (SWEMWBS) ^22, 23^
Personality	50-item International Personality Item Pool (IPIP) ^24^: Extraversion, Conscientiousness, and Emotional Stability items only
Perceived Stress	Perceived Stress Scale (PPS) 4-item version ^25, 26^
Resilience	Brief Resilience Scale (BRS) ^27^
***Questionnaires***	
COVID-19 knowledge and attitudes, health literacy *	Chicago COVID-19 Comorbidities Survey ^18^
Childcare during COVID-19 *	The Gender Division of Childcare during the COVID-19 Pandemic survey ^19^

*Items adapted for use in CovidLife

Before launching the first CovidLife questionnaire, feedback on the questionnaire was sought from collaborators and other research groups. The questionnaire was piloted on a small sample of participants and was then edited following feedback from test participants before being formally launched.

Given the sensitivity of some of the questions, and possible reservations about providing personal and sensitive information in an online study, no question required an answer. Many sensitive questions had a “prefer not to answer” option. Following feedback during the piloting phase, options to skip certain sensitive sections were added. Skip options were added to the social support, mood, and employment sections. These were introduced to enable participants to provide as much information as they felt comfortable sharing while also encouraging them to continue.

### Sample and recruitment

***CovidLife1.*** Anyone aged 18 years and over and resident in the UK could take part in CovidLife. Adults who were resident in the UK but were temporarily elsewhere in the world were also eligible. Due to the online nature of the study, individuals without access to the internet were not able to take part. Multiple methods were used to invite participants into CovidLife.

*Generation Scotland:* Generation Scotland ( www.generationscotland.org) is a family-based health study in which 23,960 adults living in Scotland aged 18 to 100 years from 6,973 families were recruited between 2006 and 2011 ^15^. Most Generation Scotland participants attended a clinic visit where biological samples were collected, physical measurements were taken, and participants completed cognitive function and mental health assessments. Prior to the clinic visit, participants answered a self-completion questionnaire assessing sociodemographic information, health and lifestyle. Participants consented to linkage with Scottish medical records. In 2015–2016, a subsample of 9,618 Generation Scotland participants took part in a detailed assessment of mental health and resilience as part of the Stratifying Resilience and Depression Longitudinally (STRADL) study ^28, 29^.

Generation Scotland participants with a known email address (n = 9,030) were invited to take part in CovidLife. The email included a link to the CovidLife1 questionnaire. Postal invites were sent to Generation Scotland participants for whom no email address was known (n = 13,766).

*General public:* Traditional media (television and radio news programs), organic social media (Facebook, Twitter, and Instagram), and targeted social media (Facebook and Instagram) were used to advertise the study to the general public. In addition to general targeting, Facebook and Instagram were also used to specifically target recruitment of male participants, those who did not have at least a bachelor degree level qualification, and those aged 18–30 years. Specific targeting of these groups was used to try and make the sample more representative of the general population.

*Other research groups:* Researchers from the Aberdeen Children of the 1950s (ACONF) study ^30, 31^ shared the CovidLife1 questionnaire link with a subsample of their volunteers via social media and email. ACONF consists of 12,150 individuals born in Aberdeen between 1950 and 1956 who completed the Aberdeen Child Development Survey when in primary school and who have been followed up in adulthood.

Two health research registers were also used to recruit participants. The Scottish Health Research Register (SHARE) includes people aged 11 and over who are interested in taking part in health research in Scotland ^32^. SHARE emailed a total of 80,000 members of the register with information about the CovidLife study and a link to take part. We also used the North West London Health Research Register ^33^ to advertise CovidLife. This register consists of adults aged 18 years and older living in North West London who consented to being contacted about health research opportunities. A total of 6,000 members of the North West London Health Research Register were sent emails inviting them to take part in CovidLife with two reminder emails sent in the following two weeks.

To start CovidLife1 potential participants use the questionnaire web link in either the email, letter, website, or social media post. Participants read through the Volunteer Information Sheet (VIS), and they gave their consent to taking part by ticking their agreement to each of the 8 consent statements (the VIS and consent are available in the Extended Data ^17^). Participants consented to be re-contacted to take part in future studies. Only after consenting were participants able to start the CovidLife1 questionnaire.

Data collection for CovidLife1 commenced on Friday 17 ^th^ April 2020 and closed to new responses on Sunday 7 ^th^ June 2020. Participants had 72 hours to complete CovidLife1 after starting it. For most of the time that CovidLife1 was open to participants, the four nations of the UK were under strict “stay at home” orders. The “stay at home” order was lifted in May (ranging from 10 ^th^ May in England to 29 ^th^ May in Scotland); however, restrictions remained in place, including limits on the number of people who could meet up outdoors, a ban on meeting people indoors, and the continued closure of non-essential businesses. A timeline of the COVID-19 restrictions during the each wave of CovidLife is available in the *Extended data* ^17^.

***CovidLife2.*** Email addresses were available for 15,256 CovidLife1 participants. These participants were emailed an invite containing a personalised link to CovidLife2. The CovidLife2 questionnaire consisted of two sections: 1) a core section, containing many of the same questions asked in CovidLife1; and 2) an optional section, consisting of new topics. These new topics incorporated some suggested by participants in the free-text question at the end of CovidLife1. They included cancelled or postponed healthcare during COVID-19, cancelled events, changes to childcare responsibilities and home-schooling during COVID-19.

Data collection for CovidLife2 began on 21 ^st^ July 2020 and closed on 16 ^th^ August 2020. Participants had 7 days to complete CovidLife2 after starting. More time was given to completing CovidLife2 because participants were sent a personalised link which allowed them to partially complete the questionnaire, stop, and return later to their saved responses by clicking back on their personalised link. Participants who had not completed CovidLife2 were sent up to two reminder emails on 31 ^st^ July 2020 (n = 7,483) and on 14 ^th^ August 2020 (n = 5,063). CovidLife2 was carried out when the COVID-19 cases in the UK were relatively low, and the restrictions in place were less strict than during CovidLife1, though some local restrictions remained in place. Most people in the UK could meet up with a small number of individuals both outdoors and indoors, non-essential shops and restaurants were open, and travel restrictions had been lifted.

***CovidLife3.*** All CovidLife1 participants who provided an email address were invited to participate in CovidLife3 (n = 15,192). Participants who did not complete CovidLife2 were still eligible to take part in CovidLife3. Slightly fewer participants were invited to CovidLife3 than were invited to CovidLife2 because some participants had inactive email addresses and others asked that we do not contact them again. The CovidLife3 invite email contained a personalised link to the CovidLife3 questionnaire.

Data collection for CovidLife3 began on 1 ^st^ February 2021 and closed on 21 ^st^ February 2021. Participants had 7 days to complete CovidLife3 after starting. A reminder email was sent to participants who had not responded to CovidLife3 between 12 ^th^ February and 15 ^th^ February 2021 (n = 6,340). CovidLife3 was carried out during another “stay at home” order, following a large increase in COVID-19 cases and deaths in the UK during December 2020 and January 2021.

### Ethical considerations

The CovidLife study was reviewed and given a favourable opinion by the East of Scotland Research Ethics Committee (Reference: 20/ES/0021, AM02, AM04, AM05, AM11).

Participants read through the VIS and they gave their consent to taking part by ticking their agreement to each of eight consent statements (VIS and consent are available in the *Extended data* ^17^). Participants consented to be re-contacted to take part in future studies. 

## Dataset description

### Demographic characteristics

***CovidLife1.*** A total of 23,118 individuals clicked on the CovidLife1 questionnaire link and were recorded as a response in Qualtrics. The following responses were removed:

1. Responses collected before the official launch time of 11:45pm on 17 ^th^ April 2020 (n = 160)
2. Responses who completed <8% of the questionnaire. Qualtrics saves responses on a given page only after the participant presses “Next”, therefore anyone who did not press “Next” on the first page of the questionnaire did not have any saved data (n = 4,006)
3. Participants who completed the questionnaire more than once were identified based on matching name and email address. Where there was >1 response per participant, the most complete or first response was retained and all others were removed (n = 380)
4. Participants aged under 18 years (n = 3)
5. Participants who did not answer any questions (n = 51).

**Figure 1.**
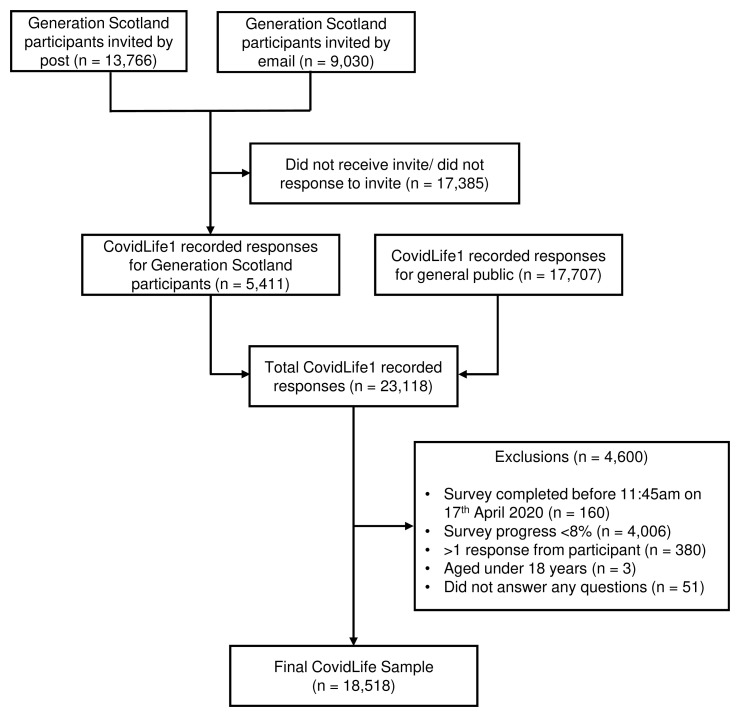
CovidLife participant flow chart.

Therefore, 18,518 participants make up the CovidLife sample. A flow chart of how we derived the CovidLife analytic sample is shown in Figure 1.

**Figure 2.**
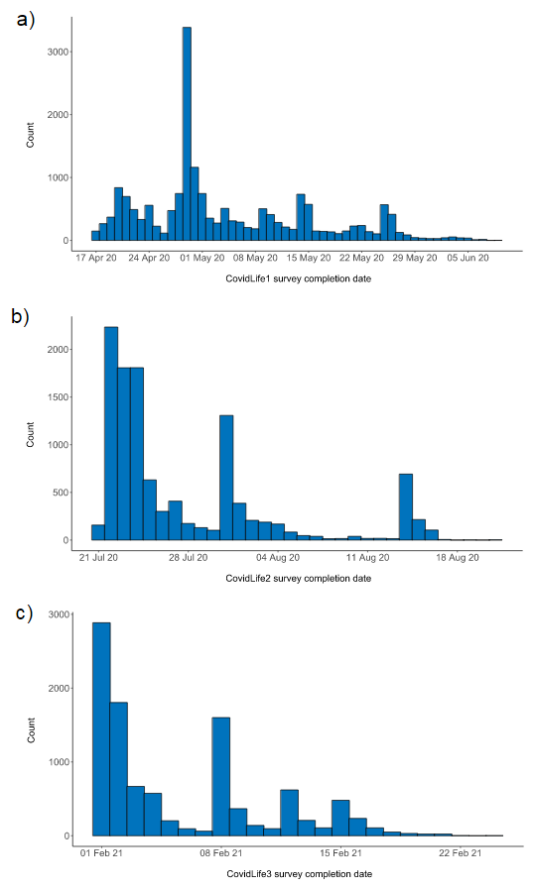
Completion dates for ( **a**) CovidLife1, ( **b**) CovidLife2, and ( **c**) CovidLife3.

**Figure 3.**
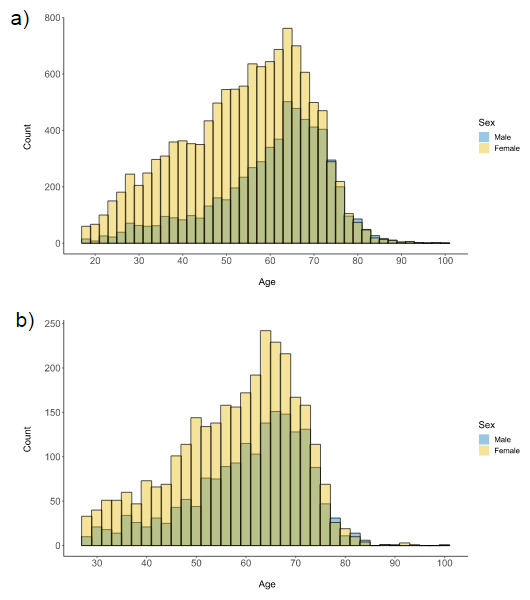
Age distribution of ( **a**) CovidLife participants, and ( **b**) Generation Scotland participants who took part in CovidLife, grouped by sex.

Figure 2 shows the number of participants who completed CovidLife on any given day during recruitment. The large peak (n = 3,386) seen on 29 ^th^ April 2020 corresponds to when SHARE invited 50,000 of their members to take part in CovidLife. Demographic and socioeconomic characteristics of CovidLife participants are reported in Table 3. The mean age of participants was 56.43 (SD = 14.35). Two-thirds were female (12,375 female, 6,016 male). The age distribution of the CovidLife sample grouped by sex is shown in Figure 3. There were more female than male participants at every age band until the mid-70s, when there were comparable, but low, numbers of male and female participants. Most participants were white (n = 16,960, 97.7%). Although CovidLife1 was open to anyone living in the UK, most (n = 16,995; 92.2%) reported living in Scotland, reflecting the use of mainly Scottish resources for recruitment (i.e., Generation Scotland, ACONF, and SHARE). The sample was highly educated, with over half (n = 8,730, 51.9%) reporting having an undergraduate degree. Over half of participants reported being employed (n = 6,937, 47.9%) or self-employed (n = 981, 6.8%), whereas 30.9% (n = 4,469) were retired.

**Table 3.** Demographic and socioeconomic characteristics reported at baseline for participants in CovidLife1, CovidLife2 and CovidLife3.

	Full sample (n = 18518)				CovidLife2 (n = 11319)				CovidLife3 (n = 10386)			
	N (%) with data		N (%) or Mean (SD)		N (%) with data		N (%) or Mean (SD)		N (%) with data		N (%) or Mean (SD)	
Age in years, mean (SD)	18308	(98.9%)	56.43	(14.35)	11296	(99.8%)	58.57	(13.25)	10358	(99.7%)	58.99	(12.90)
Age categories, n (%)	18308	(98.9%)			11296	(99.8%)			10358	(99.7%)		
18 to 29 years			987	(5.4%)			373	(3.3%)			287	(2.8%)
30 to 39 years			1798	(9.8%)			820	(7.3%)			719	(6.9%)
40 to 49 years			2567	(14.0%)			1379	(12.2%)			1241	(12.0%)
50 to 59 years			4054	(22.1%)			2558	(22.6%)			2309	(22.3%)
60 to 69 years			5528	(30.2%)			3846	(34.0%)			3649	(35.2%)
70 to 79 years			2994	(16.4%)			2072	(18.3%)			1931	(18.6%)
80 years and older			380	(2.1%)			248	(2.2%)			222	(2.1%)
Sex, n (%)	18422	(99.5%)			11319	(100.0%)			10386	(100.0%)		
Female			12375	(67.2%)			7601	(67.2%)			6954	(67.0%)
Male			6016	(32.7%)			3706	(32.7%)			3424	(33.0%)
Prefer not to answer			31	(0.2%)			12	(0.1%)			8	(0.1%)
Gender ^1^, n (%)	16452	(88.8%)			9936	(87.8%)			9127	(87.9%)		
Female			10815	(65.7%)			6516	(65.6%)			5978	(65.5%)
Male			5542	(33.7%)			3378	(34.0%)			3113	(34.1%)
Non-binary			38	(0.2%)			18	(0.2%)			17	(0.2%)
Prefer not to answer			57	(0.3%)			24	(0.2%)			19	(0.2%)
Country, n (%)	18423	(99.5%)			11319	(100.0%)			10386	(100.0%)		
Scotland			16995	(92.2%)			10740	(94.9%)			9883	(95.2%)
England			1312	(7.1%)			532	(4.7%)			467	(4.5%)
Wales			42	(0.2%)			23	(0.2%)			23	(0.2%)
Northern Ireland			41	(0.2%)			19	(0.2%)			12	(0.1%)
Elsewhere			33	(0.2%)			5	(0.04%)			1	(0.01%)
Ethnicity, n (%)	17354	(93.7%)			11094	(98.0%)			10167	(97.9%)		
White			16960	(97.7%)			10925	(98.5%)			10019	(98.5%)
Asian			124	(0.7%)			39	(0.4%)			33	(0.3%)
Black			22	(0.1%)			4	(0.04%)			1	(0.01%)
Arab			7	(0.04%)			1	(0.01%)			2	(0.02%)
Mixed			105	(0.6%)			50	(0.5%)			45	(0.4%)
Other			43	(0.2%)			21	(0.2%)			16	(0.2%)
Prefer not to answer			93	(0.5%)			54	(0.5%)			51	(0.5%)
Education, n (%)	16805	(90.7%)			10754	(95.0%)			9867	(95.0%)		
Undergraduate degree			8730	(51.9%)			5627	(52.3%)			5121	(51.9%)
No undergraduate degree			8075	(48.1%)			5127	(47.7%)			4746	(48.1%)
Employment status at baseline, n (%)	14473	(78.2%)			9171	(81.0%)			8359	(80.5%)		
Employed			6937	(47.9%)			4146	(45.2%)			3670	(43.9%)
Self-employed			981	(6.8%)			596	(6.5%)			551	(6.6%)
Retired			4469	(30.9%)			3246	(35.4%)			3046	(36.4%)
Other			2086	(14.4%)			1183	(12.9%)			1092	(13.1%)
SIMD deciles ^2^, n (%)	16915	(91.3%)			10726	(94.8%)			9873	(95.1%)		
1 – most deprived			504	(3.0%)			226	(2.1%)			201	(2.0%)
2			713	(4.2%)			387	(3.6%)			344	(3.5%)
3			861	(5.1%)			521	(4.9%)			498	(5.0%)
4			1139	(6.7%)			693	(6.5%)			632	(6.4%)
5			1302	(7.7%)			790	(7.4%)			717	(7.3%)
6			1558	(9.2%)			1002	(9.3%)			886	(9.0%)
7			2045	(12.1%)			1344	(12.5%)			1203	(12.2%)
8			2384	(14.1%)			1537	(14.3%)			1395	(14.1%)
9			2575	(15.2%)			1674	(15.6%)			1591	(16.1%)
10 – least deprived			3834	(22.7%)			2552	(23.8%)			2406	(24.4%)
SIMD Urban Rural Classification ^2^, n (%)	16915	(91.3%)			10726	(94.8%)			9873	(95.1%)		
Large Urban			7474	(44.2%)			4548	(42.4%)			4218	(42.7%)
Other Urban			3903	(23.1%)			2544	(23.7%)			2407	(24.4%)
Accessible Small Towns			1411	(8.3%)			957	(8.9%)			897	(9.1%)
Remote Small Towns			440	(2.6%)			275	(2.6%)			256	(2.6%)
Accessible Rural			2832	(16.7%)			1851	(17.3%)			1622	(16.4%)
Remote Rural			855	(5.1%)			551	(5.1%)			473	(4.8%)

^1^Question added to questionnaire on 21 ^st^ April 2020. ^2^Only available for participants providing Scottish postcode SIMD, Scottish Index of Multiple Deprivation.

For participants living in Scotland who provided a postcode, Scottish Index of Multiple Deprivation deciles were derived, which rank levels of deprivation based on the amount of employment, income, health, education, housing, access, and crime in the area where the participant lives ^34^. Scottish participants tended to live in less deprived locations, with 22.7% (n = 3,834) living in the least deprived decile. Most Scottish participants lived in urban areas (large urban n = 7,474, 44.2%; other urban n = 3,903, 23.1%).

***CovidLife2.*** In total, 11,552 participants clicked on the CovidLife2 questionnaire link. After removal of participants with <5% progress (the first point in the questionnaire where data is saved; n = 112), multiple responses from the same participants (n = 89), participants who had been removed from the CovidLife sample (n = 31), and participants who did not answer any questions (n = 1), data were available from 11,319 participants. This is 61.1% of all those in CovidLife1 and 74.2% of those invited to take part in CovidLife2. The demographic and socioeconomic characteristics of participants who took part in CovidLife2 are shown in Table 3. Generally, CovidLife2 participants were similar to CovidLife1 participants in terms of demographic and socioeconomic characteristics.

***CovidLife3.*** A total of 10,593 participants clicked on the CovidLife3 questionnaire link. The following responses were removed: responses with <6% progress (the first point in the questionnaire where data is saved; n = 118), multiple responses from the same participant (n = 61), participants who were removed from the CovidLife sample (n = 26), and participants who did not answer any questions (n = 2). Therefore, data is available from 10,386 participants. This is 56.1% of those in CovidLife1, and 68.4% of those invited to CovidLife3. In total, 9,116 participants completed all three CovidLife questionnaires (49.2% of the CovidLife sample). The demographic and socioeconomic characteristics of participants who completed CovidLife3 are reported in Table 3.

### Indicators of health

A range of general health and COVID-19-related health questions were included in CovidLife, and descriptive statistics for some of these reported at baseline are shown in Table 4. Most of the CovidLife sample reported very good (n = 7,435, 40.4%) or excellent (n = 3,053, 16.6%) general health. In total, 7.8% (n = 1,432) reported that they had been instructed to shield because they were at risk of serious illness from COVID-19. Participants self-reported whether they had a range of different physical and mental health conditions ( Table 4). One-fifth (n = 3,681, 20.2%) of participants reported having hypertension, 12.6% (n = 2,300) reported asthma, and 6.3% (n = 1,156) reported having type 2 diabetes. Nearly one-quarter (n = 4,293, 23.6%) reported having depression and 16.2% (n = 2,955) reported having anxiety.

**Table 4.** Self-reported health variables reported at baseline.

	Full sample (n = 18518)				Generation Scotland (n = 4847)			
	N (%) with data		N (%) or Mean (SD)		N (%) with data		N (%) or Mean (SD)	
General health, n (%)	18424	(99.5%)			4847	(100.0%)		
Excellent			3053	(16.6%)			966	(19.9%)
Very good			7435	(40.4%)			2248	(46.4%)
Good			5162	(28.0%)			1219	(25.1%)
Fair			2101	(11.4%)			337	(7.0%)
Poor			673	(3.7%)			77	(1.6%)
Instructed to shield, n (%)	18418	(99.5%)			4847	(100.0%)		
Yes			1432	(7.8%)			208	(4.3%)
No			16986	(92.2%)			4639	(95.7%)
COVID-19 status, n (%)	18207	(98.3%)			4823	(99.5%)		
Positive test			70	(0.4%)			12	(0.2%)
Suspected			1871	(10.3%)			375	(7.8%)
No			16266	(89.3%)			4436	(92.0%)
BMI ^1^, mean (SD)	18159	(98.1%)	27.47	(6.13)	4814	(99.3%)	26.70	(5.43)
Smoking status, n (%)	17729	(95.7%)			4760	(98.2%)		
Current smoker			1317	(7.4%)			259	(5.4%)
Former smoker			5468	(30.8%)			1499	(31.5%)
Never smoker			10944	(61.7%)			3002	(63.1%)
Self-reported health conditions, n (%)								
Asthma	18231	(98.5%)	2300	(12.6%)	4825	(99.5%)	445	(9.2%)
COPD	18231	(98.5%)	419	(2.3%)	4825	(99.5%)	87	(1.8%)
Chronic heart disease	18231	(98.5%)	413	(2.3%)	4825	(99.5%)	76	(1.6%)
Type 1 diabetes	18231	(98.5%)	324	(1.8%)	4825	(99.5%)	36	(0.7%)
Type 2 diabetes	18231	(98.5%)	1156	(6.3%)	4825	(99.5%)	215	(4.5%)
Hypertension	18231	(98.5%)	3681	(20.2%)	4825	(99.5%)	980	(20.3%)
Anxiety	18217	(98.4%)	2955	(16.2%)	4819	(99.4%)	562	(11.7%)
Depression	18217	(98.4%)	4293	(23.6%)	4819	(99.4%)	849	(17.6%)
Panic attacks	18217	(98.4%)	1219	(6.7%)	4819	(99.4%)	212	(4.4%)

^1^Calculated using self-reported height and weight. COPD, Chronic Obstructive Pulmonary Disease. BMI, Body Mass Index.

### Social and psychological measures

A range of social and psychological measures were included in CovidLife. Current anxiety and depressive symptoms were assessed in all three questionnaires using the Generalised Anxiety Disorder 7-item scale ^20^ (score range = 0–21) and Patient Health Questionnaire 9-item scale ^21^ (score range = 0–27), respectively. Subjective well-being, which was assessed using the 7-item Short Warwick-Edinburgh Mental Well-being Scale ^22, 23^ (score range = 7–35), was also measured in all questionnaires. Current life satisfaction, isolation and loneliness were each assessed using a single item in all questionnaires. Participants were asked to rate how satisfied with life they were using an 11-point scale from (0) “not at all satisfied” to (10) “extremely satisfied”. To measure isolation, participants were asked to indicate how much they felt isolated from others on an 11-point scale from (0) “not at all” to (10) “a lot”. Loneliness was assessed by asking participants how lonely they felt during the past week. Participants selected one of four options, ranging from “none or almost none of the time” to “all, or almost all of the time”. To estimate pre-pandemic life satisfaction, loneliness and isolation, CovidLife1 also asked participants to report how satisfied with life, how lonely and how isolated they felt “before COVID-19 measures were introduced (i.e., January 2020)”. In each questionnaire, a four-option question was used to assess how often participants had felt nervous or stressed because of COVID-19 in the last two weeks ^18^ (ranging from none to all of the time). Perceived stress was measured in CovidLife2 and CovidLife3 using the 4-item version of the Perceived Stress Scale ^25, 26^ (score range = 0–16).

Health literacy, resilience, and the personality traits extraversion, conscientiousness, and emotional stability were measured once. Health literacy – the ability to understand and process health information ^35^ – was measured using a 10-item self-report scale ^18^ (score range = 10–40). The 6-item Brief Resilience Scale ^27^ (score range = 1–6) was administered in CovidLife2 to measure a participant’s ability to bounce back after an adverse event. Thirty questions from the 50-item IPIP were used to measure personality ^24, 36^. Ten-items each were used to assess extraversion, consciousness, and emotional stability (score range for each personality trait = 10–50).

Summary statistics for these social and psychological variables measured in CovidLife1, CovidLife2 and CovidLife3 are reported in Table 5 and Table 6.

**Table 5.** Descriptive statistics (mean and standard deviation) for psychological and social variables.

	CovidLife1 (n = 18518)				CovidLife2 (n = 11319)				CovidLife3 (n = 10386)			
	N (%) with data				N (%) with data				N (%) with data			
	N	%	Mean	SD	N	%	Mean	SD	N	%	Mean	SD
GAD-7	16728	(90.3%)	4.79	(5.26)	10634	(93.9%)	3.65	(4.66)	9613	(92.6%)	4.67	(5.16)
PHQ-9	16391	(88.5%)	5.29	(5.60)	10434	(92.2%)	4.54	(5.20)	9444	(90.9%)	5.69	(5.67)
SWEMWBS	17712	(95.6%)	24.23	(5.12)	11112	(98.2%)	24.81	(5.03)	10138	(97.6%)	23.9	(4.99)
Extraversion	17528	(94.7%)	30.58	(8.00)	-	-	-	-	-	-	-	-
Conscientiousness	17461	(94.3%)	37.85	(6.16)	-	-	-	-	-	-	-	-
Emotional stability	17530	(94.7%)	33.55	(8.44)	-	-	-	-	-	-	-	-
Health literacy	17483	(94.4%)	37.69	(2.78)	-	-	-	-	-	-	-	-
Life satisfaction: Before COVID-19	17739	(95.8%)	7.90	(1.80)	-	-	-	-	-	-	-	-
Life satisfaction: Now	17737	(95.8%)	5.81	(2.46)	11137	(98.4%)	6.71	(2.17)	10174	(98.0%)	5.76	(2.35)
Isolation: Before COVID-19	17806	(96.2%)	1.60	(2.45)	-	-	-	-	-	-	-	-
Isolation: Now	17806	(96.2%)	6.17	(3.10)	11192	(98.9%)	3.61	(2.96)	10220	(98.4%)	5.59	(2.92)
Perceived Stress Scale	-	-	-	-	11159	(98.6%)	5.01	(3.20)	10207	(98.3%)	5.41	(3.20)
Brief Resilience Scale	-	-	-	-	11136	(98.4%)	3.56	(0.82)	-	-	-	-

GAD-7, Generalised Anxiety Disorder 7-item questionnaire; PHQ-9, Patient Health Questionnaire 9-item version; SWEMWBS, Short Warwick-Edinburgh Mental Well-being Scale.

**Table 6.** Descriptive statistics (n and percent) for psychological and social variables.

	CovidLife1 (n = 18518)				CovidLife2 (n = 11319)				CovidLife3 (n = 10386)			
	N (%) with data		N	%	N (%) with data		N	%	N (%) with data		N	%
GAD-7	16728	(90.3%)			10634	(93.9%)			9613	(92.6%)		
None to Mild			13881	(83.0%)			9444	(88.8%)			8025	(83.5%)
Moderate to Severe			2847	(17.0%)			1190	(11.2%)			1588	(16.5%)
PHQ-9	16391	(88.5%)			10434	(92.2%)			9444	(90.9%)		
None to Mild			13283	(81.0%)			8933	(85.6%)			7537	(79.8%)
Moderate to Severe			3108	(19.0%)			1501	(14.4%)			1907	(20.2%)
Loneliness: Before COVID-19	17752	(95.9%)			-	-			-	-		
None or almost none of the time			13626	(76.8%)			-	-			-	-
Some of the time			3813	(21.5%)			-	-			-	-
Most of the time			240	(1.4%)			-	-			-	-
All or almost all of the time			73	(0.4%)			-	-			-	-
Loneliness: Now	17767	(95.9%)			11164	(98.6%)			10198	(98.2%)		
None or almost none of the time			10109	(56.9%)			7980	(71.5%)			6034	(59.2%)
Some of the time			6056	(34.1%)			2708	(24.3%)			3458	(33.9%)
Most of the time			1097	(6.2%)			334	(3.0%)			504	(4.9%)
All or almost all of the time			505	(2.8%)			142	(1.3%)			202	(2.0%)
Stress because of COVID-19	17803	(96.1%)			11155	(98.6%)			10188	(98.1%)		
Never			4937	(27.7%)			4524	(40.6%)			3331	(32.7%)
Some of the time			10548	(59.2%)			5968	(53.5%)			5766	(56.6%)
Most of the time			1884	(10.6%)			564	(5.1%)			908	(8.9%)
All of the time			434	(2.4%)			99	(0.9%)			183	(1.8%)

GAD-7, Generalised Anxiety Disorder 7-item questionnaire; PHQ-9, Patient Health Questionnaire 9-item version

### Generation Scotland subsample

Of the 18,518 participants who make up the CovidLife sample, 4,847 (26.2%) were members of the Generation Scotland cohort. The demographic and socioeconomic characteristics of the Generation Scotland subsample are reported in Table 7, and health characteristics are reported in Table 4. The age distribution grouped by sex is shown in Figure 3. The age distribution for the Generation Scotland subsample was slightly older than for the CovidLife sample owing to the fact that Generation Scotland participants were all aged over 18 at recruitment (2006–2011). For this subsample, researchers can link CovidLife responses with a wealth of data collected in Generation Scotland. The number and percentage of the CovidLife sample with different types of linkable Generation Scotland data is reported in Table 8.

**Table 7.** Demographic and socioeconomic characteristics reported at baseline for Generation Scotland participants in CovidLife.

	N (%) with data		N (%) or Mean (SD)	
Age in years, mean (SD)	4844	(99.9%)	59.44	(12.12)
Age categories, n (%)	4844	(99.9%)		
18 to 29 years			43	(0.9%)
30 to 39 years			362	(7.5%)
40 to 49 years			595	(12.3%)
50 to 59 years			1107	(22.9%)
60 to 69 years			1706	(35.2%)
70 to 79 years			959	(19.8%)
80 years and older			72	(1.5%)
Sex, n (%)	4847	(100.0%)		
Female			3060	(63.1%)
Male			1787	(36.9%)
Prefer not to answer			0	(0.0%)
Gender ^1^, n (%)	4081	(84.2%)		
Female			2556	(62.6%)
Male			1521	(37.3%)
Non-binary			2	(0.05%)
Prefer not to answer			2	(0.05%)
Country of residence, n (%)	4846	(100.0%)		
Scotland			4816	(99.4%)
England			23	(0.5%)
Wales			1	(0.02%)
Northern Ireland			1	(0.02%)
Elsewhere			5	(0.1%)
Ethnicity, n (%)	4707	(97.1%)		
White			4663	(99.1%)
Asian			11	(0.2%)
Black			1	(0.02%)
Arab			1	(0.02%)
Mixed			16	(0.3%)
Other			3	(0.1%)
Prefer not to answer			12	(0.3%)
Education, n (%)	4532	(93.5%)		
Undergraduate degree			2154	(47.5%)
No undergraduate degree			2378	(52.5%)
Employment status	3812	(78.6%)		
Employed			1780	(46.7%)
Self-employed			314	(8.2%)
Retired			1314	(34.5%)
Other			404	(10.6%)
SIMD deciles, n (%)	4847	(100.0%)		
1 – most deprived			152	(3.1%)
2			195	(4.0%)
3			203	(4.2%)
4			278	(5.7%)
5			354	(7.3%)
6			398	(8.2%)
7			602	(12.4%)
8			773	(15.9%)
9			842	(17.4%)
10 – least deprived			1050	(21.7%)
SIMD Urban Rural Classification, n (%)	4847	(100.0%)		
Large Urban			2359	(48.7%)
Other Urban			1059	(21.8%)
Accessible Small Towns			319	(6.6%)
Remote Small Towns			74	(1.5%)
Accessible Rural			871	(18.0%)
Remote Rural			165	(3.4%)

^1^Question added to questionnaire on 21st April 2020. SIMD, Scottish Index of Multiple Deprivation.

**Table 8.** Number and percentage of CovidLife participants with different types of linkable Generation Scotland data.

	N	%
Detailed phenotyping at baseline (2006–2011)	4739	(25.6%)
Linkage with Scottish medical records using CHI number	4845	(26.2%)
Genotype data	4359	(23.5%)
DNA methylation data	2701	(14.6%)
STRADL mental health questionnaire (2015–2016)	4031	(21.8%)
STRADL clinic visit (2015–2016)	634	(3.4%)

CHI, Community Health Index; STRADL, Stratifying Resilience and Depression Longitudinally

The Generation Scotland baseline assessment (2006–2011) consisted of a pre-clinical questionnaire and a clinic visit ^14^. The pre-clinical questionnaire collected information on sociodemographic characteristics, medical history, family history, mood, and health behaviours. During the clinic visit, physical measurements including height, weight, blood pressure, and ankle-brachial pressure index were taken. Psychological measurements included tests of cognitive function, personality, psychological distress, and screening for emotional and psychiatric problems using the structured clinical interview for the Diagnostic and Statistical Manual IV disorders. Biological samples were also collected. A total of 4,739 (25.6%) CovidLife participants had data collected as part of the Generation Scotland baseline clinical assessment.

Samples collected during the Generation Scotland baseline have been used to derive genotype and DNA methylation data. A total of 4,359 (23.5%) CovidLife participants have genotype data and 2,701 (14.6%) currently have DNA methylation data available. At the time of writing, DNA methylation data is being processed for approximately 10,000 additional Generation Scotland participants, and proteomic data will also be available on a sub-sample of Generation Scotland participants in the future.

Most Generation Scotland participants consented to their study data being linked with Scottish medical records, using their Community Health Index numbers. Generation Scotland study data has been linked with a range of different routinely collected health datasets, including morbidity records (SMR 01, 02, 04, 06, 11), GP records, death records, prescribing data, and routine lab tests. This includes regular updates on COVID-19 testing, diagnoses and vaccination records. Data from 4,845 (26.2%) CovidLife participants can be linked with medical records.

In the 2015–2016 Generation Scotland mental health follow-up study (STRADL), a subsample of Generation Scotland participants were sent a questionnaire assessing demographic information, medical history, resilience, self-reported psychiatric symptoms, psychological distress, threatening experiences, and coping strategies. In total, 4,031 (21.8%) CovidLife participants completed the STRADL questionnaire. Some of these participants also attended a STRADL clinic visit, where participants underwent brain magnetic resonance imaging, blood samples were collected, and tests of mental health and cognitive functioning were administered. In total, 634 (3.4%) CovidLife participants attended the STRADL clinic visit.

More detailed information on the measures collected in Generation Scotland and STRADL are available elsewhere ^14, 28, 29^.

### Strengths and limitations

CovidLife has a number of strengths, one being the size of the cohort, which stands at over 18,000 participants. The cohort is well characterised, including the collection of demographic, health, social, psychological, and economic information. COVID-19 specific information on infections, symptoms, compliance and opinions of the handling of COVID-19 in the UK was also collected. In particular, this study included many psychological measures, most of which have been assessed on three occasions throughout the COVID-19 pandemic. This means that CovidLife can be used to understand how people were feeling and behaving early in the COVID-19 pandemic, and how this has changed over time.

Three waves of CovidLife have been carried out, and these waves coincide with important milestones in the COVID-19 pandemic in the UK (see the CovidLife timeline in *Extended data* ^17^). CovidLife1 was carried out during the first UK “stay at home” order shortly after COVID-19 cases had peaked (in wave 1) in the UK. CovidLife2 took place when the rates of COVID-19 infections and deaths were relatively low and restrictions had eased considerably. During this time, most people living in the UK were able to meet up with family and friends both outdoors and indoors, though some places were under tighter local restrictions. Many people had returned to work, cafés, restaurants and retail were open, and schools were planning to fully reopen after the summer holidays.

COVID-19 cases and deaths began to rise in autumn and winter 2020, and another stay at home order was implemented in all four UK nations by 4 ^th^ January, 2021. COVID-19 cases peaked in early January 2021. CovidLife3 data collection took place in February 2021 during this stay at home order. COVID-19 cases and deaths were higher in this period of lockdown compared to the first. However, the UK mass COVID-19 vaccination programme began on 8 ^th^ December 2020 and was well underway during the second lockdown ^37^. By collecting data during these important milestones in the COVID-19 pandemic, CovidLife can be used to understand the health, well-being and behaviour of people in the UK, and how these change as the restrictions have eased and tightened, and as individual circumstances have changed throughout the pandemic.

The ability to link responses with data collected in Generation Scotland for 4,847 participants is one of the key advantages of CovidLife. Responses can be linked with a wide range of demographic, health, and lifestyle information, collected many years before the pandemic. Genetic and biological sample data can also be linked with responses. Importantly, CovidLife responses can be linked with medical records. Regular releases of NHS Scotland health record data make it possible to examine both retrospective and prospective associations with health outcomes.

The CovidLife questionnaires were designed to harmonise with other research studies in the UK and around the world. Many of the measures included in the CovidLife study align with those used in other longitudinal population health studies. Subsequently, it has been possible to collaborate with other research groups to investigate the mental health impact of COVID-19. Using data from CovidLife and the Avon Longitudinal Study of Parents and Children ^7^, anxiety and depression during the early stages of the pandemic were found to be greater in young participants, women, those with pre-existing physical and mental health conditions, and those with lower socioeconomic status. A recent pre-print ^38^ combined the results from 12 longitudinal studies (n = 59,482), including the Generation Scotland subsample of CovidLife, to test whether pre-pandemic psychological distress was associated with healthcare, economic, and housing disruption during the COVID-19 pandemic. Higher pre-pandemic psychological distress was associated with increased odds of healthcare disruption, loss of employment and income, and reduced working hours or being furloughed, but it was not associated with housing disruption during the pandemic ^38^. Another pre-print using data from over 65,000 individuals in 12 longitudinal studies, including CovidLife, found that healthcare disruption during COVID-19 was greater in female participants, older people, ethnic minorities, and those from more disadvantaged social classes ^39^.

There are some limitations to CovidLife. Questionnaires were administered online and therefore this study was restricted to those with internet access. The sample consists mostly of people who have previously shown interest in health research, either by being a member of a health study or by signing up to a health research register. Therefore the sample was not fully representative of those living in the UK, and consisted mostly of participants who were relatively highly educated, white, and from less deprived areas. Although there was a large number of Scottish participants, the other three nations of the UK were less well represented. Like all longitudinal studies, CovidLife suffers from attrition. In total, 49.2% of those in CovidLife1 completed all three CovidLife questionnaires. Due to these limitations, any conclusions drawn from CovidLife data may not generalise to all groups in society.

To conclude, the CovidLife study allows us to understand: 1) the mental health, well-being and behaviour of people living in the UK during the COVID-19 pandemic; 2) how these vary according to demographic, health and economic circumstances; and 3) how mental health, well-being and behaviour change over time as the COVID-19 pandemic progresses and as mitigation measures ease and tighten. The subsample of Generation Scotland participants in CovidLife enables the investigation of both pre-pandemic predictors of health and well-being during COVID-19 and the long-term health consequences of the pandemic. Researchers can apply to access the CovidLife and Generation Scotland data to investigate the determinants, correlates, and consequences of health and well-being during COVID-19.

## Data availability

### Underlying data

CovidLife and Generation Scotland data are available to researchers through managed access. Non-identifiable data will be made available to approved researchers in the UK and internationally.

Researchers wanting to access the CovidLife data can apply using the CovidLife Access Request Form, available in the *Extended data* ^17^. Once completed, this form should be emailed to access@generationscotland.org.

Researchers wanting to link CovidLife data with Generation Scotland data can apply for access to the Generation Scotland data using the standard Generation Scotland Access Request Form, available in the *Extended data* ^17^. Once completed, this form should be emailed to access@generationscotland.org.

Up to date information about how to apply to access CovidLife and Generation Scotland data is available on the Generation Scotland website: http://www.generationscotland.org/for-researchers/access

Generation Scotland’s withdrawal policy allows participants to request that their data no longer be available for research. Therefore when data is released, the sample size for CovidLife and Generation Scotland may vary slightly than that reported here.

### Extended data

Zenodo: Extended Data for "CovidLife: A resource to understand mental health, well-being and behaviour during the COVID-19 pandemic in the UK”. https://doi.org/10.5281/zenodo.4967815 ^17^.

The project contains the following extended data:

- 2021-06-15_CovidLife1_Questionnaire.docx (CovidLife1 questionnaire)
- 2021-06-15_CovidLife2_Questionnaire.docx (CovidLife2 questionnaire)
- 2021-06-15_CovidLife3_Questionnaire.docx (CovidLife3 questionnaire)
- 2021-06-17_CovidLife_VIS_Consent.docx (CovidLife volunteer information sheet and consent form)
- 2021-06-17_CovidLife_Timeline_v1.0.tiff (Timeline of the COVID-19 restrictions in the UK during CovidLife data collection)
- CovidLife_Access_Request_Form_V3.1_March _2021.docx (CovidLife Data Access Request Form)
- Generation_Scotland_Access_Request_Form_V1.2_March_2021.docx (Generation Scotland Data Access Request Form)
- 2021-06-17_STROBE_checklist_CovidLife_DataNote_v1.0.docx (Completed STROBE checklist)

Data are available under the terms of the Creative Commons Attribution 4.0 International license (CC-BY 4.0).
