# Supplementary material for: CovidLife: a resource to understand mental health, well-being and behaviour during the COVID-19 pandemic in the UK
Source: Wellcome Open Res. 2021 Jul 7;6:176. [Version 1] doi: 10.12688/wellcomeopenres.16987.1 (PMC10884595; doi:10.12688/wellcomeopenres.16987.1)
Supplement: Peer review history

# Reviewer report: Reviewer response for version 1

**DOI:** 10.21956/wellcomeopenres.18755.r74085

**Published:** 2024-02-21

**Authors:** Joel W Hughes

**Affiliations:** 1 Department of Psychological Sciences, Kent State University, Kent, Ohio, USA

Thank you for the opportunity to review this article describing the CovidLife resource. The data collected as part of the CovidLife study should yield valuable information and insights for years. The dataset is large, and the first wave of data was collected very early in the Covid-19 pandemic. As the article was published in 2021, my comments should be considered suggestions and commentary.

 I do not recommend any changes, but increasing the value of subsequent reports may be possible if researchers can employ a few analytic techniques.

 The data were collected using Qualtrics survey software. It may be possible to examine the data for quality using recommendations that were recently published:

 Belliveau, J., & Yakovenko, I. (2022). Evaluating and improving the quality of survey data from panel and crowd-sourced samples: A practical guide for psychological research. *Experimental and Clinical Psychopharmacology*, *30*(4), 400.

 Eyal, P., David, R., Andrew, G., Zak, E., & Ekaterina, D. (2021). Data quality of platforms and panels for online behavioral research. *Behavior Research Methods*, 1-20.

 For example, it is possible to check for “speeders” who may not have provided reliable data (i.e., completing the survey in less than 1/3 the median time). For some measures, checking for “straight lining” (i.e., selecting the same option for many consecutive items) may reveal any participants using a response style that could have provided invalid data. Given that respondents may have been motivated to provide accurate information, I suspect there will be very little invalid data.

 Some missing data are inevitable. Depending on the analysis, it may be possible to use multiple imputation procedures to preserve the sample size.

 Many of the respondents resided in Scotland. Is it possible to weight the data of the subsample residing in Scotland using known population values for Scotland? Perhaps the representativeness of the sample could be improved in this manner.

 When interpreting the findings of future reports, is there anything about Scottish culture that researchers should know?

 Is there a bibliography of studies published (or planned) using the CovidLife dataset?

 Is it possible to calculate a response rate for the respondents from the Generation Scotland study?  

 The CovidLife is understandably linked to the Covid-19 pandemic. The enthusiasm of some researchers and journal reviewers may wane as that global infectious disease pandemic recedes into history. That is a sentiment I do not share because the COVID-19 pandemic was just one example of a shared stressor. There will be outbreaks of diseases in the future, and understanding how people cope, respond, and are affected should help us prepare.

Are sufficient details of methods and materials provided to allow replication by others?

Yes

Is the rationale for creating the dataset(s) clearly described?

Yes

Are the datasets clearly presented in a useable and accessible format?

Yes

Are the protocols appropriate and is the work technically sound?

Yes

Reviewer Expertise:

Clinical Health Psychology

I confirm that I have read this submission and believe that I have an appropriate level of expertise to confirm that it is of an acceptable scientific standard.

# Reviewer report: Reviewer response for version 1

**DOI:** 10.21956/wellcomeopenres.18755.r46181

**Published:** 2021-10-27

**Authors:** Margarita Panayiotou

**Affiliations:** 1 Manchester Institute of Education, The University of Manchester, Manchester, UK

I appreciate the invitation to review this work. The current project clearly involves a massive undertaking by the team and this paper provides a very clear and detailed guide of the data. The tables and figures are particularly useful. I make some suggestions below, which hopefully the authors will find useful:

1. It is great that the questionnaire pack was piloted with a smaller sample ahead of data collection. Could the authors provide some more information about that sample? 
2. What was the target recruitment based on (i.e. how were these gaps in the sample identified)?
3. Suggestion for a table: I wonder if it would be useful to have a table with the data collection time points and under each time point note the key COVID-19 events. 

Dataset description:

1. It would be useful to have the % of the sample alongside the Ns. 
2. Were there any differences in terms of demographics between those agreeing to participate in time 2 and 3 and those that dropped out?

Social & psychological measures:

1. A justification is needed about the 11-point scale for quality of life/loneliness items.
2. I'd suggest renaming the first column of Table 3 to CovidLife1.
3. Were the psychometric properties of measures assessed (e.g. internal consistency)? 

Are sufficient details of methods and materials provided to allow replication by others?

Yes

Is the rationale for creating the dataset(s) clearly described?

Yes

Are the datasets clearly presented in a useable and accessible format?

Yes

Are the protocols appropriate and is the work technically sound?

Yes

Reviewer Expertise:

adolescent mental health, assessment and psychometrics

I confirm that I have read this submission and believe that I have an appropriate level of expertise to confirm that it is of an acceptable scientific standard.
